# *Macleaya cordata* Extract Ameliorates DSS-Induced Ulcerative Colitis via Inhibiting M1 Macrophages and Reshaping Gut Microbiota

**DOI:** 10.3390/antiox15101344

**Published:** 2026-10-16

**Authors:** Changhua Zhang, Yong Zhang

**Affiliations:** 1 College of Veterinary Medicine, Gansu Agricultural University, Lanzhou 730070, China; 13672667399@163.com; 2 Gansu Key Laboratory of Animal Generational Physiology and Reproductive Regulation, Lanzhou 730070, China

**Keywords:** *Macleaya cordata* extract (MCE), sanguinarine (SAN), chelerythrine (CHE), ulcerative colitis, gut microbiota, macrophage polarization

## Abstract

*Macleaya cordata*, which is widely distributed in nature, exhibits pronounced anti-inflammatory, antioxidant, and anti-colitis potential. However, its therapeutic mechanism on DSS-induced ulcerative colitis (UC) remains unknown; therefore, we aimed to evaluate the protective effect of *Macleaya cordata* extract (MCE) on UC and investigate its active monomer component. In a mouse colitis model induced by DSS, MCE markedly alleviated pathological characteristics. 16S rRNA sequencing indicated a significant reduction in the relative abundance of pathogenic *Streptococcus*, while increasing that of beneficial genera *Dubosiella* and *Akkermansia*. Furthermore, flow cytometry analysis indicated the protective role of MCE on DSS-induced macrophage polarization in the colon (32.6% reduction in M1 macrophage compared to the DSS group; 5.3% increase in M2 macrophage compared to the DSS group) and spleen tissue (36.8% reduction in M1 macrophage compared to the DSS group; 22.3% increase in M2 macrophage compared to the DSS group). In addition, we found that sanguinarine (SAN) (27.9% reduction in body weight compared to the DSS group; *p* < 0.01) exhibited a superior anti-colitis effect compared to chelerythrine (CHE) (19.8% reduction in body weight compared to the DSS group) by inhibiting NF-κB activation and ROS production than CHE in LPS-induced RAW 264.7 macrophages. In conclusion, MCE ameliorates UC by reshaping gut microbiota and suppressing M1 polarization.

## 1. Introduction

Ulcerative colitis (UC) is a chronic inflammatory bowel disease of unknown etiology, clinically marked by a relapsing-remitting course and histologically characterized by diffuse, continuous mucosal inflammation [1,2]. The inflammatory process commonly begins in the rectum and spreads proximally to involve contiguous segments of the colon [3,4]. An imbalance in the gut microbiota and disrupted immune system homeostasis have been identified as contributing factors in the etiology of UC [5]. Macrophages are thought to be the key modulator and effector cell in the host response to foreign invasion [6]. Dysregulated macrophage function is governed by multiple inflammatory signaling pathways. Preclinical evidence indicated that altered macrophage polarization has been found in UC patients, mediating a shift between immune homeostasis and intestinal microbiota dysbiosis [7,8]. Macrophage polarization is broadly categorized into pro-inflammatory M1 and anti-inflammatory M2 phenotypes [9]. Classically activated M1-type macrophages amplify local inflammation through various inflammatory signaling pathways, especially the nuclear factor-kappa B (NF-κB) pathway [10,11]. Therefore, targeting M1 macrophages represents a promising therapeutic strategy for UC [12,13]. Recent insights suggest that the NF-κB signaling pathway, which is mediated by TLR4, is activated in the colonic tissue from UC patients [14]. Moreover, the NF-κB signaling pathway-mediated colonic inflammatory response is known to promote pro-inflammatory mediator production (such as TNF-α, IL-6, NO), which exacerbates cellular dysfunction and oxidative stress, ultimately leading to colonic tissue damage [15,16,17].

While conventional pharmacotherapy and surgery remain the cornerstone of treatment for human UC, concerns regarding high costs and adverse side effects have generated interest in medicinal plant extracts as novel, reliable, and safe alternatives [18,19]. *Macleaya cordata* is a traditional herb that has a worldwide distribution, including in China, North America, and Europe [20]. Historically, *Macleaya cordata* has been utilized both as a feed supplement in livestock production, targeting ailments including swine dysentery, white scours, edema disease, paratyphoid, and porcine infectious gastroenteritis, and as a prominent herbal medicine in traditional Chinese practice. In the latter context, it has been extensively employed to manage bruises, chronic ulcers, rheumatic complaints, scabies, and various other cutaneous affections [21,22,23]. In addition, a growing body of evidence has reported that *Macleaya cordata* extract (MCE) exhibits diverse pharmacological activities, ranging from insecticidal and antimicrobial to anti-inflammatory, antioxidant, and antitumor activities [24,25,26]. Phytochemical analyses have revealed that MCE contains a variety of bioactive compounds, including alkaloids, amino acids, coumarins, volatile oils, saponins, steroids, flavonoids, and carbohydrates [27,28,29]. Of particular interest are the alkaloid components, which have been the subject of intensive research owing to their significant therapeutic potential [30,31]. Among these, sanguinarine (SAN) and chelerythrine (CHE), the two major representatives of the quaternary benzo[c]phenanthridine alkaloid (QBA) class, are abundantly present in MCE leaves and have been widely studied for their multifaceted biological activities [24,32].

This study was designed to investigate the protective effect of *Macleaya cordata* extract (MCE) on 2.5% dextran sulfate sodium (DSS)-induced colitis in mice, using 5-aminosalicylic acid (5-ASA) as a reference compound. The experimental results reveal that MCE treatment significantly alleviates the severity of DSS-evoked colonic injury. Mechanistically, this beneficial action appears to be associated with the restoration of gut microbiota homeostasis and the rebalancing of M1/M2 macrophage polarization patterns. Further analysis revealed that the monomer SAN, a main constituent of MCE, also exhibits protective effects against colitis by suppressing M1-type macrophage activation. Mechanistic studies have demonstrated that the anti-colitis effect of SAN may be exerted through the inhibition of NF-κB-mediated inflammatory activation and the activation of the Nrf2-Keap1-HO-1 signaling pathway, thereby enhancing antioxidant stress responses.

## 2. Materials and Methods

### 2.1. Chemicals and Materials

*Macleaya cordata* extract (0.375 g/100 g formulation, batch No. 250733) was obtained from Hunan Micolta Bioresource Co., Ltd. (Changsha, China) (manufacturing date: 2 July 2025). Sanguinarine (SAN, >95% pure) (CAS:2447-54-3), and Chelerythrine (CHE, >95% pure) (CAS:34316-15-9) was supplied by Hunan Micolta Biological Resources Co. Ltd. (Changsha, China).

### 2.2. Animal Experiment

For the establishment of the murine colitis model, a total of 25 female C57BL/6J specific pathogen-free (SPF) mice, aged 6–8 weeks and weighing 18 ± 2 g (supplied by Hunan SJA Laboratory Animal Co., Ltd., SCXK 2024-009, Changsha, China), were randomly assigned to various treatment groups (n = 5). These included a vehicle-treated control group, a 2.5% DSS group receiving vehicle only, two dosage levels of Macleaya cordata extract (50 and 100 mg/kg) administered intragastrically for eight consecutive days, a 5-ASA group (100 mg/kg, intragastrically for 8 days), as well as two dose groups each for SAN (1 and 10 mg/kg) and CHE (1 and 10 mg/kg), all given via the same intragastric route over the same duration.

During both the acclimation and the experimental phases, the animals were provided with food and water ad libitum and housed under a 16 h light/dark cycle. All husbandry and experimental protocols were conducted in accordance with the guidelines approved by the Animal Care and Ethics Committee of Gansu Agricultural University (Approval number: GSAU-Eth-VMC-2025-038, Approval Date: 14 April 2025). At the end of the study, the mice were humanely sacrificed by intraperitoneal injection of tribromoethanol (Aladdin, China).

Daily inspections were performed on all experimental animals, and body weight measurements were taken at the same time each day throughout the study. In addition, the clinical progression of colitis was monitored via the daily calculation of the disease activity index (DAI), which integrates three parameters: stool consistency, fecal occult or visible blood (including rectal bleeding), and the percentage of body weight reduction relative to baseline, all of which reflect the animals’ response to DSS exposure.

### 2.3. 16s rRNA Sequencing

To evaluate the impact of MCE, SAN, and CHE on gut microbiota population, fecal samples from C57BL/6J mice were analyzed via 16s rRNA gene sequencing targeting the V3–V4 hypervariable region. Sequencing was performed on the Illumina NovaSeq PE250 platform (Illumina, Inc., San Diego, CA, USA). Library preparation followed standard protocols for metagenomic analysis. Microbial community composition was assessed to determine α-diversity and compare taxonomic profiles.

### 2.4. Splenic and Colonic Macrophages Polarization

To assess the effects of MCE, SAN, and CHE on splenic and colonic macrophages, spleens or colon obtained from C57BL/6 mice were first dissected into small pieces and then digested with 10% fetal bovine serum (FBS; ExCell Bio, Shanghai, China) RPMI 1640 medium containing collagenase IV (1.5 mg/mL), and DNase I (0.05 mg/mL) for 40 min at 37 °C with constant shaking (200 rpm). Following digestion, macrophage populations were purified by centrifugation on a discontinuous Percoll density gradient. Subsequently, 1.5 × 10^5^ single cells were stained with a panel of antibodies, including CD16/32 (Biolegend, San Diego, CA, USA, 156603), CD45 (Biolegend, 103116), CD80 (Biolegend, 104705), CD11b (Biolegend, 101212), CD206 (Biolegend, 141706), and Brilliant CD68 (Biolegend, 137017). Finally, the stained cells were acquired and analyzed on a Beckman Coulter flow cytometer.

### 2.5. RAW 264.7 Cells Polarization

The RAW 264.7 murine macrophage cell line was obtained from the China Center for Type Culture Collection (CCTCC, Shanghai, China). The cells were routinely cultured in 10% FBS Dulbecco’s Modified Eagle Medium (DMEM; Gibco, Carlsbad, CA, USA), and the cell line was passaged no more than 15 times to ensure the stability of its phenotypic characteristics.

Upon completion of the stimulation, the cells were harvested and labeled with CD80 (Biolegend, 104705) and CD206 (Biolegend, 141706). Finally, fluorescence intensity was measured using a Beckman Coulter flow cytometer.

### 2.6. MTT Assays

To evaluate the cytotoxicity of MCE, SAN, and CHE, RAW 264.7 were treated with MCE, SAN, and CHE for 24 h. Following the exposure period, MTT reagent was added and the plates were incubated for 4 h. Finally, the absorbance of the samples was measured at 490 nm using a microplate spectrophotometer.

### 2.7. NO Production Assays

The cells were then exposed to MCE, SAN, and CHE at the designated dosages for 4 h, followed by a 16 h challenge with 100 ng/mL LPS, a potent inflammatory stimulus. Nitric oxide (NO) levels were determined using the Griess reagent assay (OD540 nm).

### 2.8. Western Blot Analysis

Following treatment, the cells were harvested and lysed in RIPA buffer (P0013B; Beyotime, Beijing, China) on ice for 30 min. The blocked membranes were then probed with primary antibodies against inducible nitric oxide synthase (iNOS, #13120), phosphorylated NF-κB p65 (P-NF-κB p65, #8242), total NF-κB p65 (T-NF-κB p65, #3033), and GAPDH (#5174) (all from Cell Signaling Technology, Danvers, MA, USA). Finally, the protein bands were developed using an enhanced chemiluminescence (ECL) reagent (SQ201, Epizyme, Shanghai, China), and the resulting signals were recorded using the MiniChemi^®^ 610 chemiluminescent imaging system (Sinsage, Beijing, China).

### 2.9. Measurement of TNF-α and IL-6 by ELISA

To assess inflammatory cytokine secretion, TNF-α and IL-6 levels in RAW 264.7 cell supernatants were measured with commercial ELISA kits (4A Biotech, Beijing, China) per the manufacturer’s instructions. Absorbance was then recorded at 450 nm using a microplate reader (BMG LABTECH, Offenburg, Germany).

### 2.10. Molecular Docking Analyses and Molecular Dynamics (MD) Simulation

The 2D structures of SAN were converted into 3D conformations using Chem3D software (Version 26.0). Highly complementary protein keap1 was selected from the Protein Data Bank through structural alignment. Subsequently, these proteins were prepared by removing water molecules and phosphate groups in PyMOL (Version 3.1.) Molecular Operating Environment (MOE) 2019 was utilized for ligand energy minimization, protein preprocessing, and binding site detection. Molecular docking simulations were performed using MOE, and the resulting complexes were visualized with PyMOL and Discovery Studio.

Molecular dynamics (MD) simulations of the SAN-Keap1 complex were conducted in Gromacs 2022. The system was constructed with the AMBER99SB (Amber 99 force field) force field and solvated in a TIP3P water box, followed by a 100 ns production simulation at 298 K. Energy minimization was first performed using the steepest descent algorithm, and the system was subsequently equilibrated under NVT and NPT ensembles. The resulting trajectories were analyzed using the g_mmpbsa tool to calculate binding free energies.

### 2.11. Statistical Analysis

For statistical evaluation, an unpaired two-tailed Student’s t-test was employed to compare two experimental groups, whereas two-way ANOVA with Bonferroni’s multiple-comparison correction was applied when more than two groups were involved. The resulting *p*-values are presented with asterisks to denote significance levels relative to the overall group comparisons. All data visualization and statistical testing were conducted with GraphPad Prism (version 8.0.2).

## 3. Results and Discussion

### 3.1. The Protective Effect of MCE on DSS-Induced Mouse Colitis

**Figure 1:**
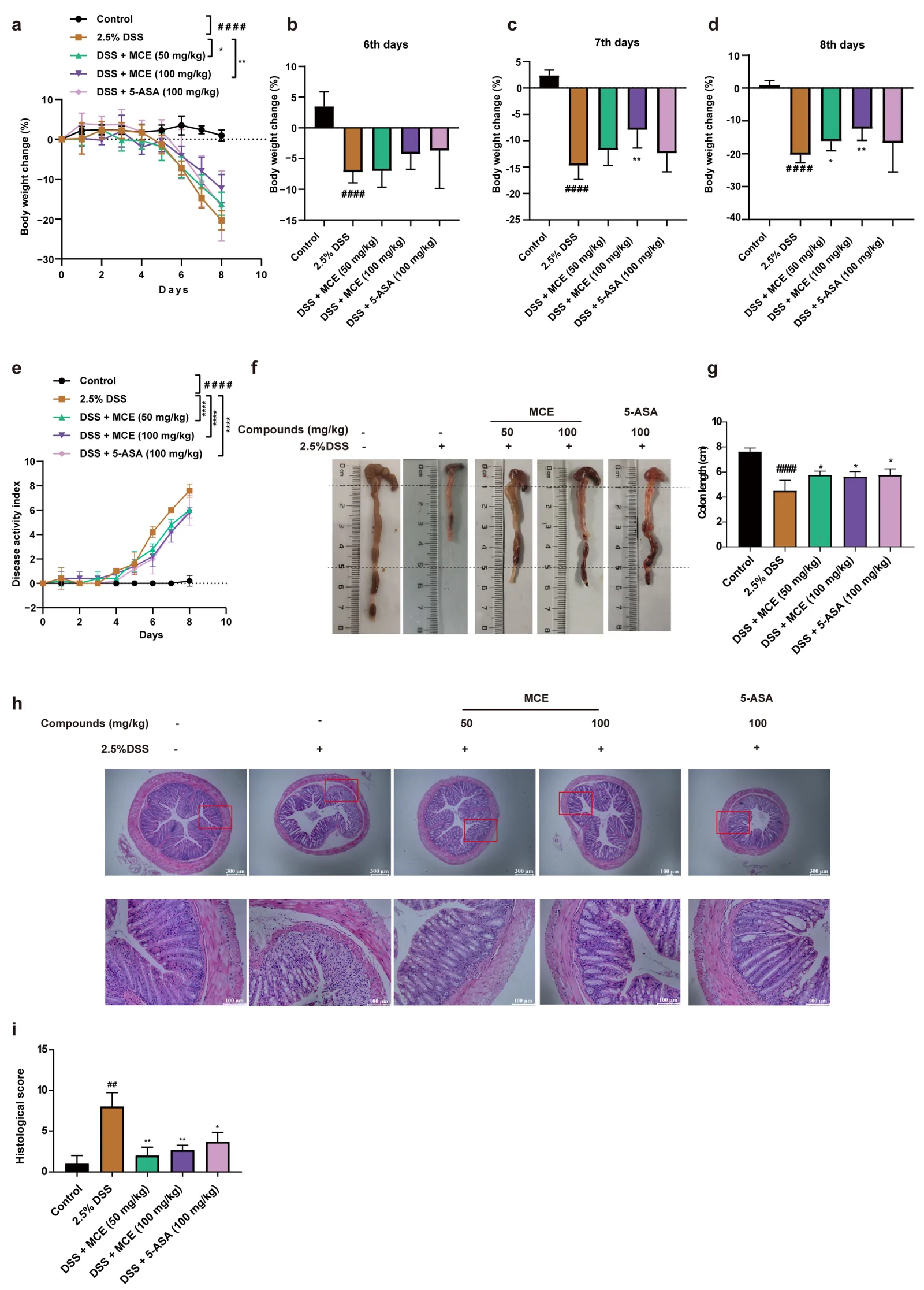
The protective effect of MCE on DSS-induced mice colitis. (**a**–**d**) The body weight changes, (**e**) DAI increased (n = 5), and (**f**,**g**) colon length shortening in mice were measured. (**h**,**i**) Hematoxylin and eosin (H&E), with scale bars set at 500 μm (top) and 100 μm (bottom) (n = 5). Data were expressed as mean ± SD. * *p* < 0.05, ** *p* < 0.01, **** *p* < 0.0001. ## *p* < 0.01, #### *p* < 0.0001.

Although *Macleaya cordata* is a traditional herb with diverse biological activities, the therapeutic effects of its extract (MCE) and its active monomeric components on UC remain to be elucidated [33,34]. A UC mouse model was established via DSS administration to explore the potential therapeutic activity of *Macleaya cordata* extracts (MCE). As shown in Figure 1a–g, 2.5% DSS treatment induced obvious pathological symptoms, including body weight loss, increased disease activity index (DAI) scores, and colon length shortening, indicating a successfully established mouse colitis model. To test the protective effect of MCE, colitis mice were orally administered 50 and 100 mg/kg MCE. 100 mg/kg 5-ASA was used as a reference compound. Compared with the DSS-induced mice, MCE treatment significantly suppressed body weight loss (50 mg/kg MCE reduced body weight by 22.7% vs. DSS; 100 mg/kg MCE reduced body weight by 40.2%) and reduced diarrhea, bloody stools, and DAI scores in a dose-dependent manner, with the most pronounced effect observed at 100 mg/kg (Figure 1a–e). Importantly, MCE demonstrated superior protective efficacy compared to the reference compound 5-ASA, with a more pronounced reduction in DSS-induced body weight loss.

Mice exposed to 2.5% DSS were observed to show a significantly reduced colon length (*p* < 0.0001), which is also an important pathological change in ulcerative colitis (Figure 1f,g). Whereas MCE-treated colitis mice were markedly protected against DSS-induced colon shortening (27.4% increase compared to DSS group; *p* < 0.05), with an effect comparable to that of 5-ASA (29.1% increase compared to DSS group; *p* < 0.05) (Figure 1f,g). Consistently, the histopathological analysis of the colon showed that DSS-induced colitis mice revealed serious pathological changes, including notable epithelial disruption, atrophied colonic crypts, disordered muscle fiber arrangement, and obvious inflammatory infiltration (Figure 1h,i). After MCE treatment, the colonic epithelium remained intact, the structure of colonic crypts was intact, the muscle fibers were arranged neatly, and the inflammatory infiltration was reduced (Figure 1h,i).

### 3.2. MCE-Treatment Reshapes the Gut Microbiota in 2.5% DSS-Induced Mouse Colitis

**Figure 2:**
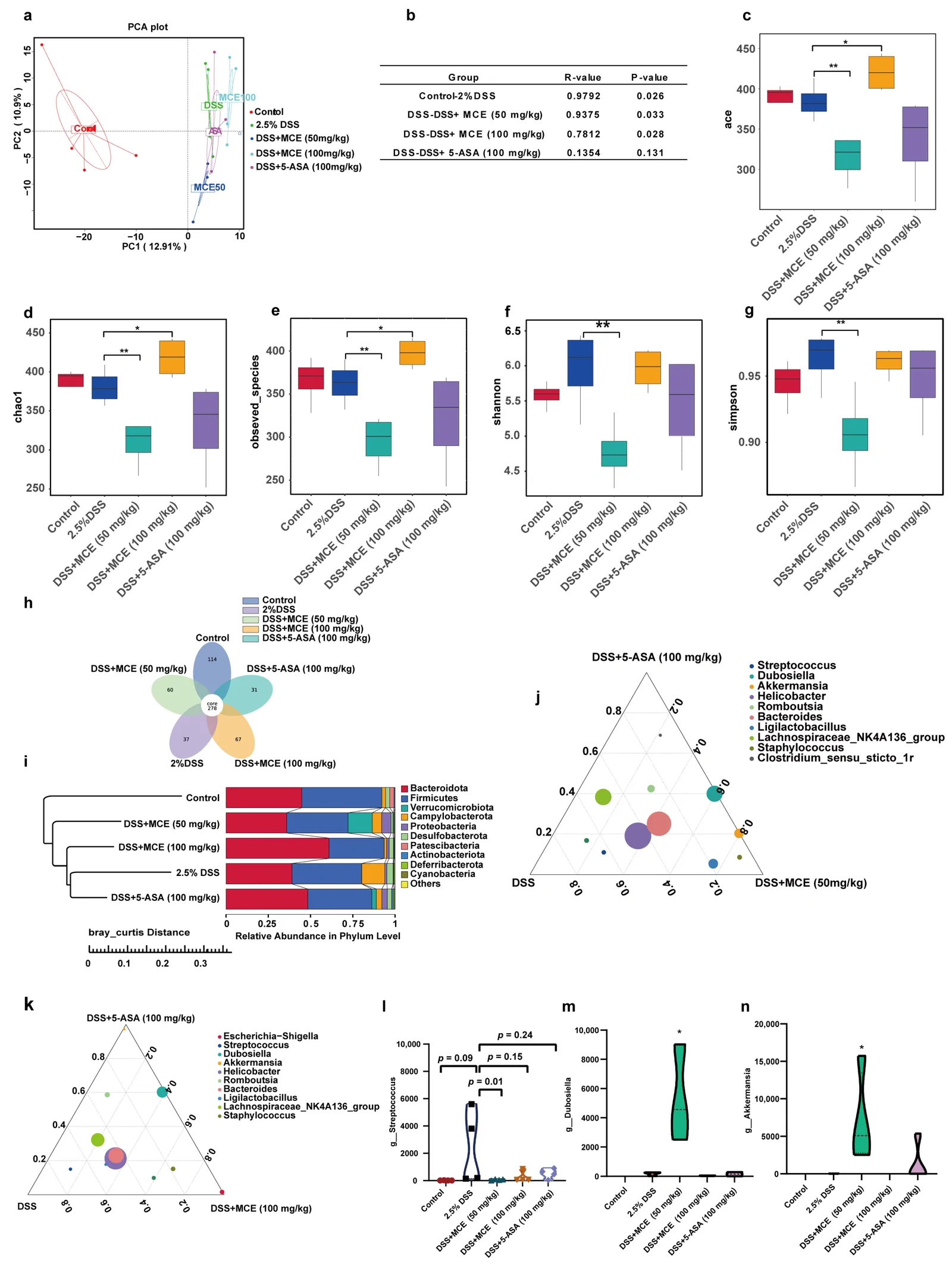
MCE treatment reshapes the gut microbiota in 2.5% DSS-induced mouse colitis. To explore the gut microbiota, 16S rRNA sequencing of the colonic microbiota was performed on fecal samples from C57BL/6J mice in five groups: control, 2.5% DSS, DSS + MCE (50 mg/kg), DSS + MCE (100 mg/kg), and DSS + 5-ASA (100 mg/kg) (n = 4 per group). (**a**) Principal component analysis (PCA) plots (**b**) and the corresponding permutational multivariate analysis of variance (Adonis test) were used to assess β-diversity (n = 4). (**c**–**g**) α-diversity analysis was performed based on the ace (**c**), chao1 (**d**), observed-species (**e**), and Shannon indices (**f**), and Simpson (**g**) (n = 4). (**h**) Venn diagram of target bacteria in the control group, 2.5% DSS, DSS + MCE (50 mg/kg), DSS +MCE (100 mg/kg), and DSS + 5-ASA (100 mg/kg) groups, respectively. (**i**) Heatmap showing the top 10 microbial phyla clustered by UPGMA. (**j**,**k**) The ternary phase diagram further showed the top 10 microbial genera based on UPGMA clustering. (**l**–**n**) The intestinal microbiota composition of *Streptococcus* (**l**), *Dubosiella* (**m**), and *Akkermansia* (**n**) among experimental groups at the genus level. * *p* < 0.05, ** *p* < 0.01.

Ulcerative colitis is closely associated with the composition of gut microbiota, which maintains the stability of the intestinal mucus layer [35,36]. To explore whether the protective effect of MCE is linked to its role in gut microbiota composition, mouse fecal samples, including the control (vehicle), DSS, DSS + MCE, and DSS + 5-ASA groups, were collected and subjected to 16S rRNA technology. When we investigated the composition of gut microbiota by sample beta-diversity differences, principal component analysis (PCA) was performed. Visually, the ordination of mice 16S rRNA composition showed a clear separation between control and DSS groups (ANOSIM, R = 0.9782, *p* = 0.026) (Figure 2a,b). Notably, 100 mg/kg MCE treatment (Anosim, R^2^ = 0.7812, *p* = 0.028) and 50 mg/kg MCE treatment (ANOSIM, R^2^ = 0.9375, *p* = 0.033) showed greater beta-diversity differences compared to that of 100 mg/kg dose of 5-ASA (ANOSIM, R^2^ = 0.1354, *p* = 0.131) (Figure 2a,b).

The α-diversity, including ACE, Chao1, observed species, Shannon, and Simpson, was further applied to describe within-group species richness and evenness. Significant increases were detected in 16S rRNA ACE, Chao1, and observed species indices between the DSS group and the DSS + MCE groups (100 mg/kg), while no significant change occurred in Shannon or Simpson indices (Figure 2c–g). Additionally, 50 mg/kg MCE reduced both gut microbiota richness and evenness (Figure 2c–g). This result indicated that the oral supplementation of 100 mg/kg MCE primarily facilitates the recruitment and restoration of microbial richness.

Further analysis of the gut bacterial composition at the phylum level showed that Bacteroidetes, Firmicutes, and Campylobacterota were the predominant phyla in the DSS-induced group. In contrast, the 50 mg/kg MCE group was predominantly colonized by *Bacteroidetes*, *Firmicutes*, and *Verrucomicrobiota*, whereas the 100 mg/kg MCE group was dominated by *Bacteroidetes*, *Firmicutes*, and *Desulfobacterota* (Figure 2h,i). Analysis of gut bacterial composition at the genus level revealed that pathogenic genera (*Streptococcus*) were enriched following 2.5% DSS induction (97.4% reduction compared to DSS group; *p* < 0.01), whereas beneficial *Dubosiella* and *Akkermansia* were enriched after oral administration of 50 mg/kg MCE (Figure 2j,l). These beneficial genera were also predominant in the 100 mg/kg 5-ASA-treated group (Figure 2k). However, the relative abundance of *Dubosiella* and *Akkermansia* was significantly higher in the 50 mg/kg MCE-treated group than in the 100 mg/kg 5-ASA-treated and MCE-treated groups (Figure 2m,n). *Dubosiella* and *Akkermansia*, two recently identified potential probiotic genera, are critical for mitigating detrimental immune responses in human UC [37,38]. Collectively, our results demonstrate that 50 mg/kg MCE significantly reduced the relative abundance of pathogenic *Streptococcus* while increasing that of beneficial genera *Dubosiella* and *Akkermansia* in DSS-induced murine colitis.

### 3.3. MCE Inhibits M1 Polarization in DSS-Induced Mice Colitis

**Figure 3:**
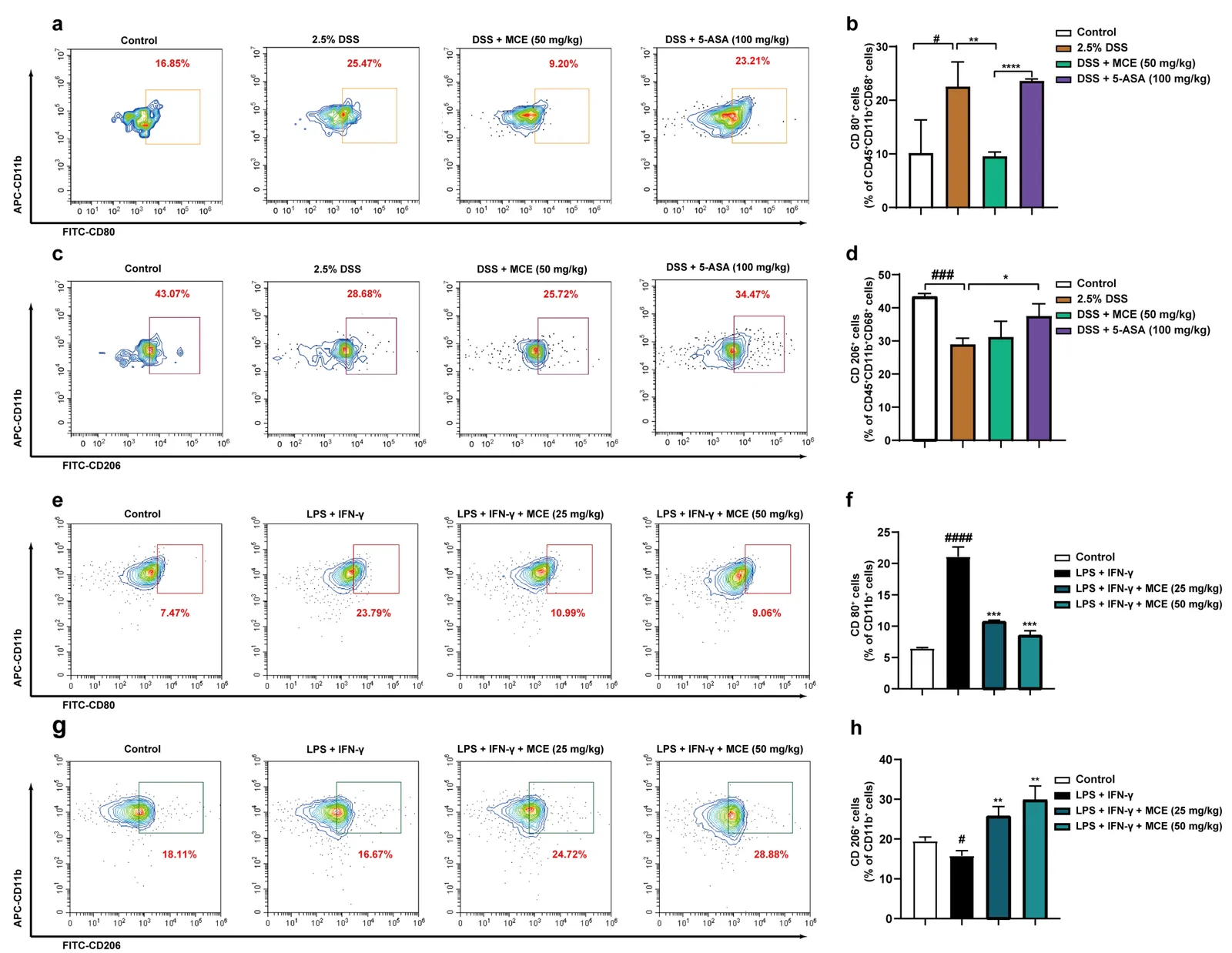
MCE inhibits M1 polarization in DSS-induced mice colitis. (**a**) Mean fluorescence intensity (MFI) of CD80 and (**c**) CD206 in colonic lamina propria cells from DSS-treated mice was measured by flow cytometry. (**b**) The populations of M1^+^ cells (CD45^+^CD11b^+^CD68^+^CD80^+^) and (**d**) M2^+^ cells (CD45^+^CD11b^+^CD68^+^CD206^+^) were also quantified. (**e**) For in vitro experiments, MFI of CD11b, CD80, and (**g**) CD206 was also assessed by flow cytometry. (**f**) The proportions of M1-like (CD11b^+^CD80^+^) and (**h**) M2-like (CD11b^+^CD206^+^) cells were determined. All group comparisons were performed using unpaired, two-tailed Student’s *t*-tests. * *p* < 0.05, ** *p* < 0.01, *** *p* < 0.001, **** *p* < 0.0001; # *p* < 0.05, ### *p* < 0.001, #### *p* < 0.0001.

Given the crucial role of beneficial bacterial genera in regulating macrophage activation [39,40], we next evaluated the impact of orally administered 50 mg/kg MCE on macrophage polarization in the colons of DSS-induced colitis mice. Flow cytometry analysis indicated that the proportion of M1^+^ cells (CD45^+^CD11b^+^CD68^+^CD86^+^) was significantly increased, whereas M2^+^ cells (CD45^+^CD11b^+^CD68^+^CD206^+^) were significantly decreased in DSS-induced colonic tissues, indicating that DSS induced a shift in macrophage polarization from the anti-inflammatory M2-like to the pro-inflammatory M1-like (Figure 3a–d). Consistently, in contrast to the DSS group, the proportion of M2^+^ cells (CD45^+^CD11b^+^CD68^+^CD206^+^) was not significantly altered in the 50 mg/kg MCE-treated group (5.3% increase in M2 macrophage compared to the DSS group). However, the M1 macrophage proportion (CD45^+^CD11b^+^CD68^+^CD86^+^) was markedly reduced in the 50 mg/kg MCE-treated group (32.6% reduction in M1 macrophage compared to DSS group) (Figure 3a–d). Our results suggested that 50 mg/kg MCE primarily influences M1 polarization against DSS-induced mice colitis.

Meanwhile, the macrophage polarization of MCE on LPS + IFN-γ-induced RAW 264.7 macrophage polarization was further investigated. In comparison to the LPS + IFN-γ-induced RAW 264.7 group, MCE reduced the M1 macrophage proportion (CD11b^+^CD86^+^) and increased the M2 macrophage proportion (CD11b^+^CD206^+^) (Figure 3e–h). Our results showed MCE inhibited M1-type macrophage activation by regulating macrophage polarization in vitro and in vivo.

**Figure 4:**
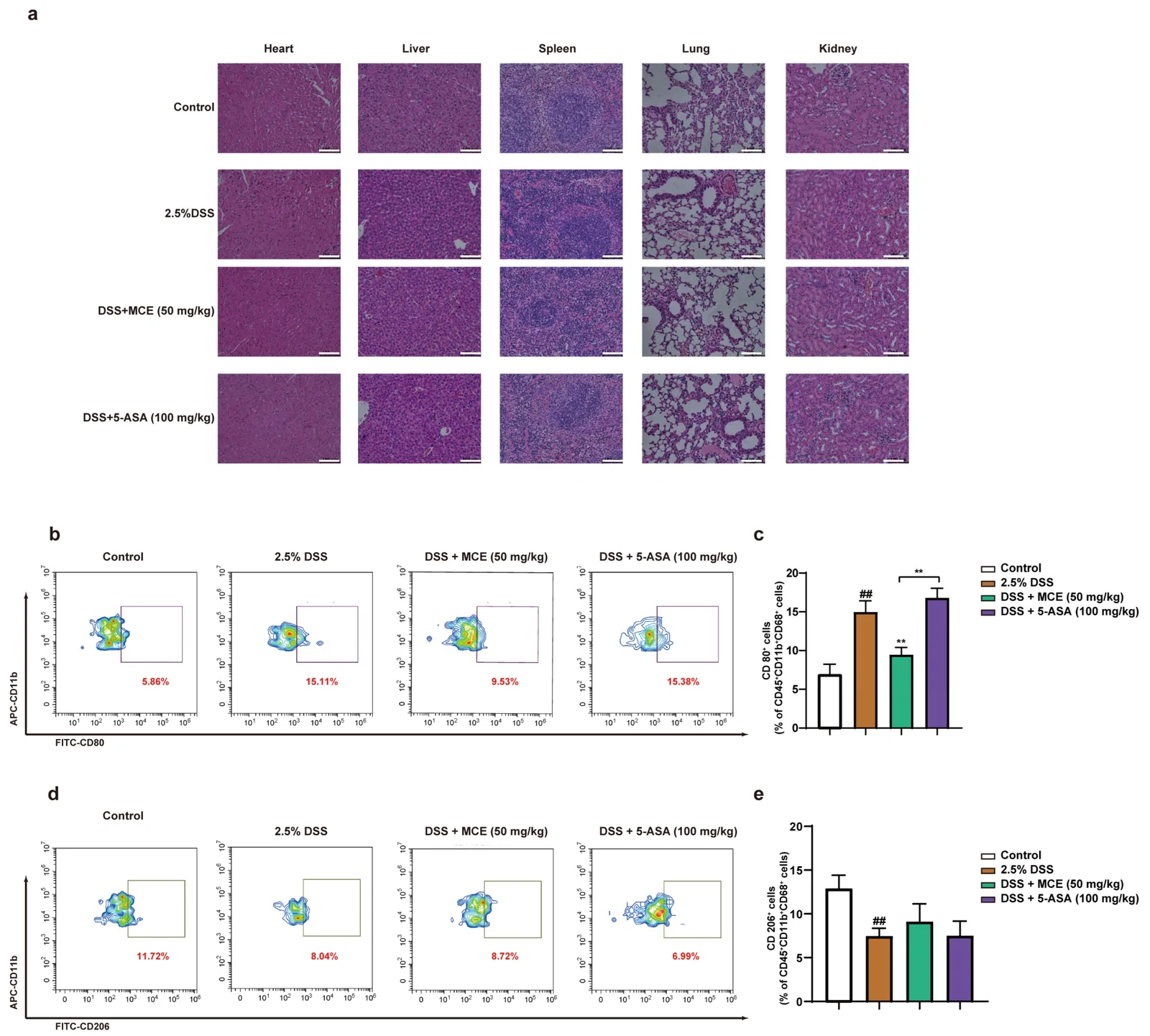
The biosafety profile of MCE in mice. (**a**) After the experimental period, H&E staining of the mice’s major organs from the MCE-treated group was performed. Scale Bar = 100 μm. (**b**) Flow cytometric analysis was conducted on splenic tissues isolated from DSS-induced mice to determine the mean fluorescence intensity of CD80 and (**d**) CD206, as well as the proportions of (**c**) M1-type (CD45^+^CD11b^+^CD68^+^CD80^+^) and (**e**) M2-type (CD45^+^CD11b^+^CD68^+^CD206^+^) macrophage populations. All data presented are representative of three independent experiments. For group comparisons, unpaired, two-tailed Student’s *t*-tests were applied, and the significance levels are indicated as follows: ** *p* < 0.01; ## *p* < 0.01.

We further assessed the biosafety of 50 mg/kg MCE by H&E staining of the major organs of MCE-treated mice, including the heart, liver, spleen, lung, and kidney (Figure 4a). No significant damage was observed in the MCE-treated heart, liver, spleen, lung, and kidney compared to the control group at the end of the experimental period, indicating that 50 mg/kg MCE effectively reduced colitis without causing toxic effects. Splenic dysfunction is a common extra-intestinal manifestation of ulcerative colitis [41]. The effect of MCE on splenic macrophage polarization was further examined. Notably, the proportion of M1^+^ cells (CD45^+^CD11b^+^CD68^+^CD86^+^) was significantly increased, whereas M2^+^ cells (CD45^+^CD11b^+^CD68^+^CD206^+^) were significantly decreased in DSS-induced splenic tissues, indicating DSS-induced shifted macrophage polarization in DSS-treated splenic cells. Treatment with 50 mg/kg MCE significantly reduced the DSS-induced increase in the proportion of M1 macrophages (36.8% reduction in M1 macrophage compared to the DSS group, *p* < 0.01) (Figure 4b–e). However, the M2 macrophage proportion showed no statistically significant change in MCE-treated colitis mice (22.3% increase in M2 macrophage compared to the DSS group, *p* > 0.05). Our results implied the promising inhibitory effect of 50 mg/kg MCE on DSS-induced pro-inflammatory M1 macrophage activation (Figure 4b–e).

### 3.4. The Protective Effect of SAN and CHE on DSS-Induced Mice Colitis

**Figure 5:**
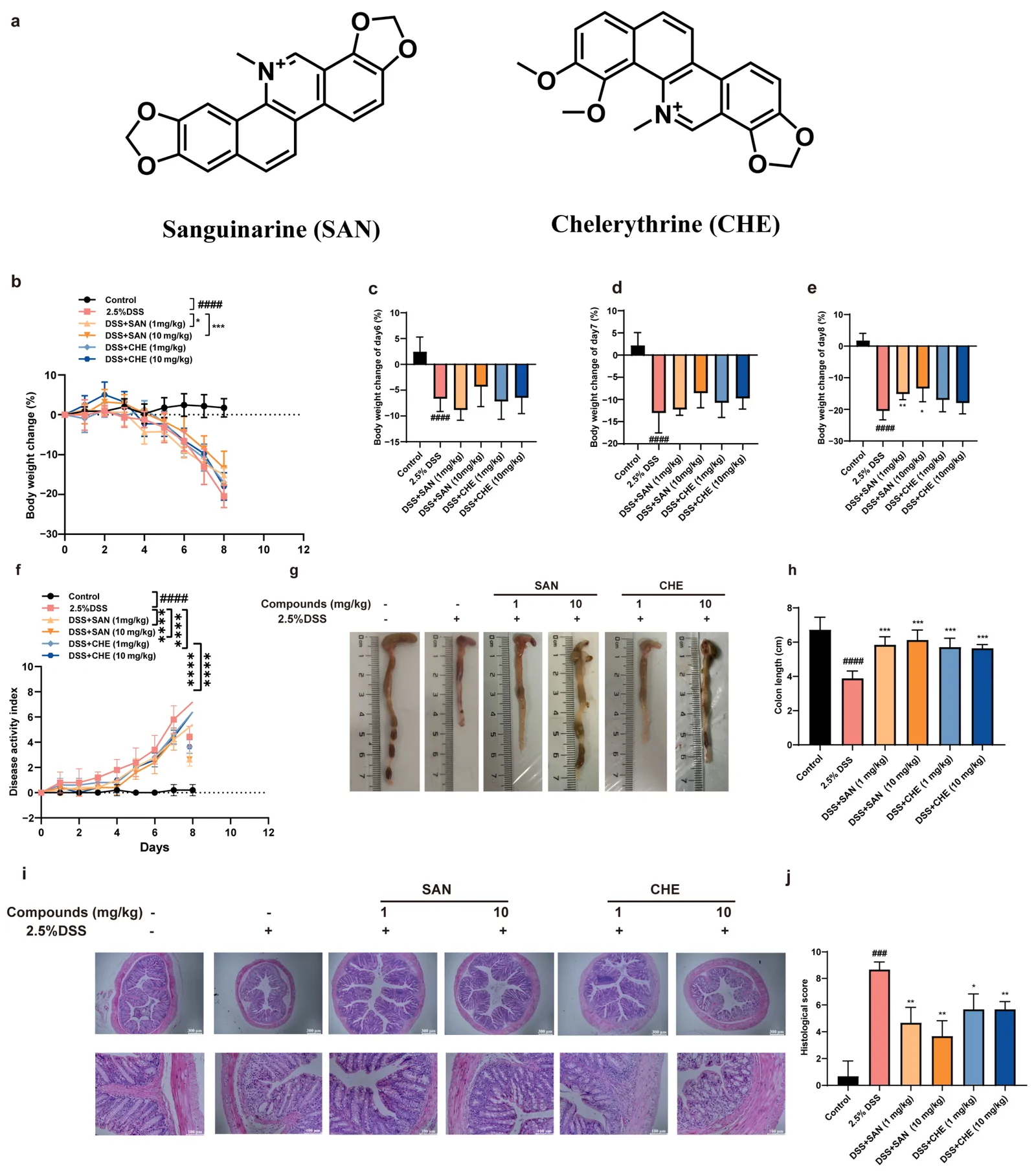
The protective effect of SAN and CHE on DSS-induced mice colitis. (**a**) The structure of Sanguinarine (SAN) and Chelerythrine (CHE). (**b**–**e**) The body weight changes, (**f**) Disease activity index (DAI) increase (n = 5), and (**g**,**h**) colon length shortening in mice were measured. (**i,j**) H&E staining. Scale bar, 500 μm (top panels), 100 μm (bottom panels). Data were expressed as mean ± SD. * *p* < 0.05, ** *p* < 0.01, *** *p* < 0.001, **** *p* < 0.0001. ### *p* < 0.001, #### *p* < 0.0001.

Given the benefit of MCE administration in reducing DSS-induced mice colitis, we further studied the effect of the main components of MCE, including sanguinarine (SAN) and chelerythrine (CHE) (Figure 5a). Mice were administered by continuous gavage of the indicated concentrations of SAN and CHE for 8 days. Administration of CHE (19.8% reduction in body weight compared to DSS group; *p* > 0.05) did not mitigate DSS-induced body weight loss, whereas SAN (27.9% reduction in body weight compared to DSS group; *p* < 0.01) administration significantly alleviated this effect (Figure 5b,e). Both SAN and CHE significantly prevented the increase in DSS-induced disease activity index (DAI) scores (Figure 5f). Furthermore, treatment with these compounds markedly attenuated colon shortening compared to the DSS-treated group (58.8% increase compared to the DSS group; *p* < 0.001) (Figure 5g,h). Notably, the body weight in the SAN group (10 mg/kg) was greater than that in the CHE group at the same dosage, indicating a stronger protective effect of SAN against DSS-induced mice colitis (Figure 5b–e). Consistently, histological analysis further indicated obvious alleviation of epithelial disruption, atrophied colonic crypts, disordered muscle fiber arrangement, and obvious inflammatory infiltration in the colon in response to 10 mg/kg SAN treatment, but not 10 mg/kg CHE treatment (Figure 5i,j). Collectively, these results indicated that SAN showed more pronounced attenuation of clinical colitis pathological symptoms and colonic damage compared to CHE.

### 3.5. The Effect of SAN on M1 Microphage Polarization on DSS-Induced Mice Colitis

**Figure 6:**
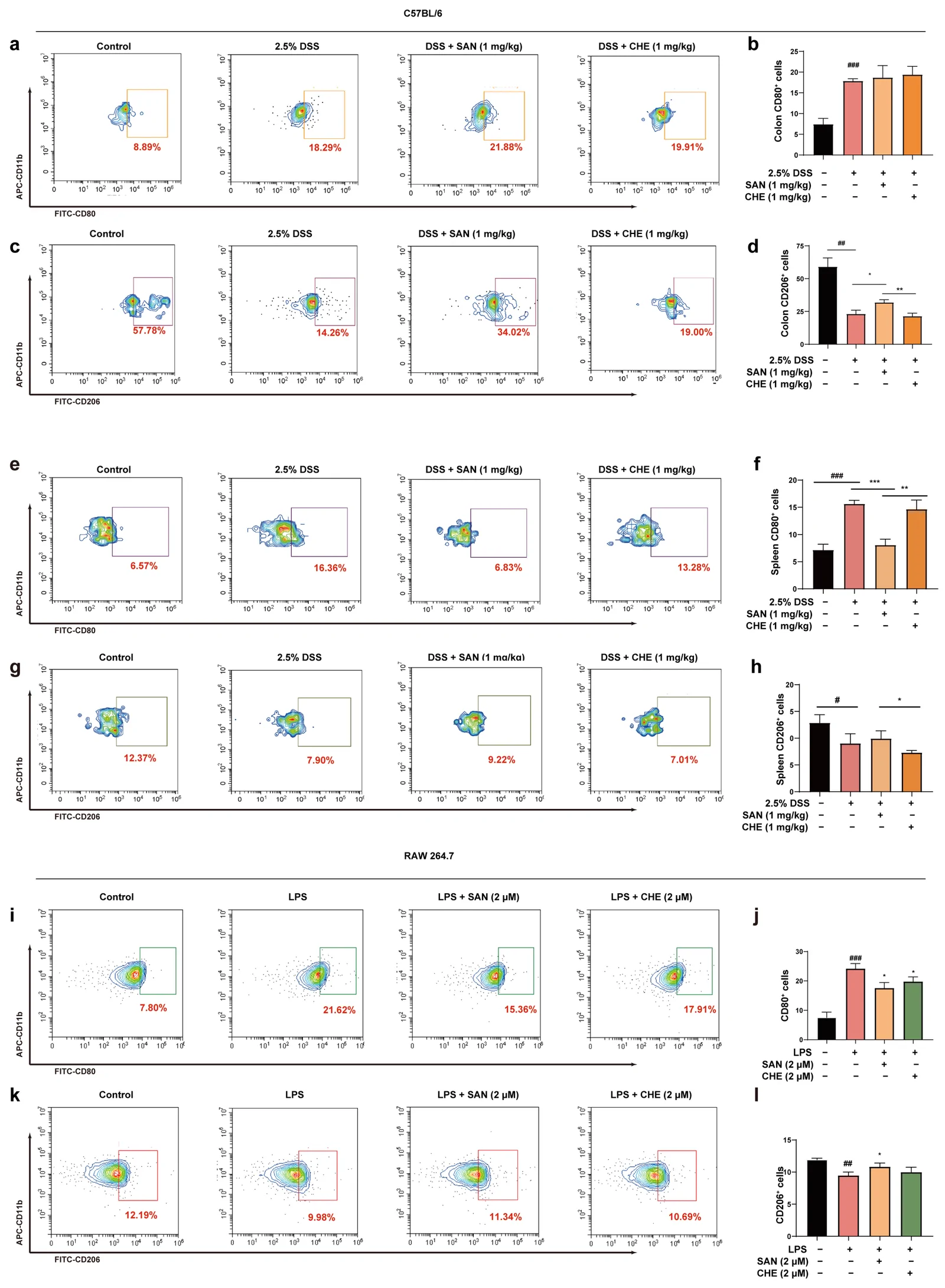
The effect of SAN on M1 microphage polarization in DSS-induced mice colitis. (**a**) The MFI of CD80 and (**c**) CD206 was measured in DSS-induced colonic lamina propria. (**b**) M1-type (CD45^+^CD11b^+^CD68^+^CD80^+^) and (**d**) M2-type (CD45^+^CD11b^+^CD68^+^CD206^+^) macrophages were quantified. (**e**) In splenic tissues from DSS-treated mice, the MFI of CD80 and (**g**) CD206 was assessed, along with the proportions of (**f**) M1- (CD45^+^CD11b^+^CD68^+^CD80^+^) and (**h**) M2-type (CD45^+^CD11b^+^CD68^+^CD206^+^) macrophages. (**i**) For in vitro studies, the MFI of CD11b, CD80, and (**k**) CD206 was also determined, as well as the percentages of (**j**) M1-like (CD11b^+^CD80^+^) and (**l**) M2-like (CD11b^+^CD206^+^) cells. All data presented are representative of three independent experiments. For group comparisons, unpaired, two-tailed Student’s *t*-tests were applied, and the significance levels are indicated as follows: * *p* < 0.05, ** *p* < 0.01, *** *p* < 0.001; # *p* < 0.05, ## *p* < 0.05, ### *p* < 0.001.

The results of our study demonstrated that MCE supplementation substantially promoted the enrichment of the favorable gut commensals *Dubosiella* and *Akkermansia*, as reflected by their increased relative abundances in the treated groups. Based on these findings, we further evaluated the effects of its bioactive constituents, SAN and CHE, on the abundance of these taxa. As shown in Figure S1a–d, CHE but not SAN treatment increased the relative abundance of *Dubosiella* and *Akkermansia*. We further investigated the effect of SAN and CHE on DSS-induced mouse colonic macrophage polarization (Figure 6a–h) and LPS + IFN-γ-induced RAW 264.7 macrophages (Figure 6i–l). Our results indicated that DSS shifted M2 macrophages to M1 macrophages both in the colon (Figure 6a–d) and spleen tissues (Figure 6e–h). Notably, 1 mg/kg sanguinarine (SAN) effectively inhibited M1 macrophage polarization in both the in vivo (Figure 6a–h) and in vitro experimental models (Figure 6i–l). This effect is consistent with the macrophage suppression mediated by *Macleaya cordata* extract (MCE). These results indicate that SAN (1 mg/kg) is a promising anti-colitis compound, consistent with the effects of MCE (Figure S2).

**Figure 7:**
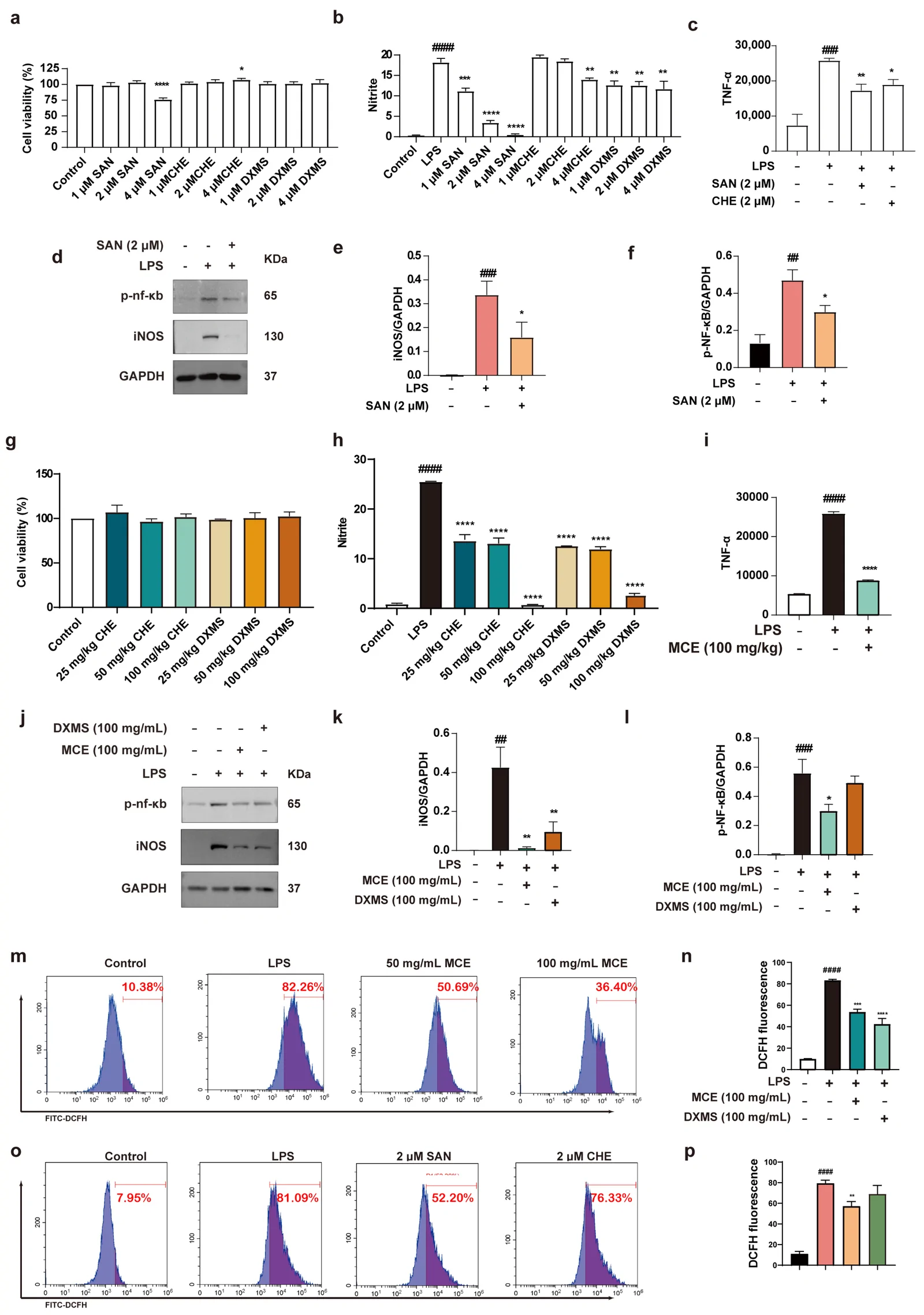
The effect of SAN on LPS-induced RAW 264.7 inflammatory response and oxidative stress. (**a**) The cytotoxicity of SAN, CHE, and dexamethasone (DXMS). (**b**) NO production was assessed via the Griess reaction. (**c**) TNF-α production. (**d**–**f**) p-NF-κB and iNOS expression, with GAPDH as a loading control. (**i**) Cytotoxicity of MCE and DXMS was determined by MTT assay. (**j**) Anti-inflammatory effects of MCE and DXMS were evaluated by the Griess reaction. (**k**) Inhibitory effect of MCE on LPS-induced TNF-α production. (**l**–**n**) p-NF-κB and iNOS expression in MCE- and DXMS-treated cells, with GAPDH as a loading control. (**o**,**p**) Inhibitory effect of MCE on LPS-induced ROS production was analyzed by flow cytometry. (**g**,**h**) Inhibitory effects of SAN and CHE on LPS-induced ROS production were assessed under the same conditions. For group comparisons, unpaired, two-tailed Student’s *t*-tests were applied, and the significance levels are indicated as follows: * *p* < 0.05, ** *p* < 0.01, *** *p* < 0.001, **** *p* < 0.0001; ## *p* < 0.01, ### *p* < 0.001, #### *p* < 0.0001.

To further verify the effect of SAN on M1-type macrophages, an LPS-induced RAW 264.7 macrophage model was established, and the pro-inflammatory mediator (NO) was detected. Our results showed that at 2 μM, both SAN and CHE significantly reduced NO levels (Figure 7a,b), while TNF-α also displayed a decreasing tendency (Figure 7c). However, 2 μM SAN showed a more pronounced effect on NO inhibition compared to CHE (Figure 7b), indicating SAN exhibited greater anti-inflammatory efficacy. Meanwhile, exposure to 2 μM SAN substantially downregulated the protein levels of iNOS and p-NF-κB, suggesting that SAN effectively blocks the iNOS/NF-κB signaling cascade (Figure 7d–f). In parallel, MCE also demonstrated a strong inhibitory action against the same pathway, without detectable cytotoxic effects (Figure 7i–n).

### 3.6. The Effect of SAN on Nrf2/HO-1 Signaling Pathway

**Figure 8:**
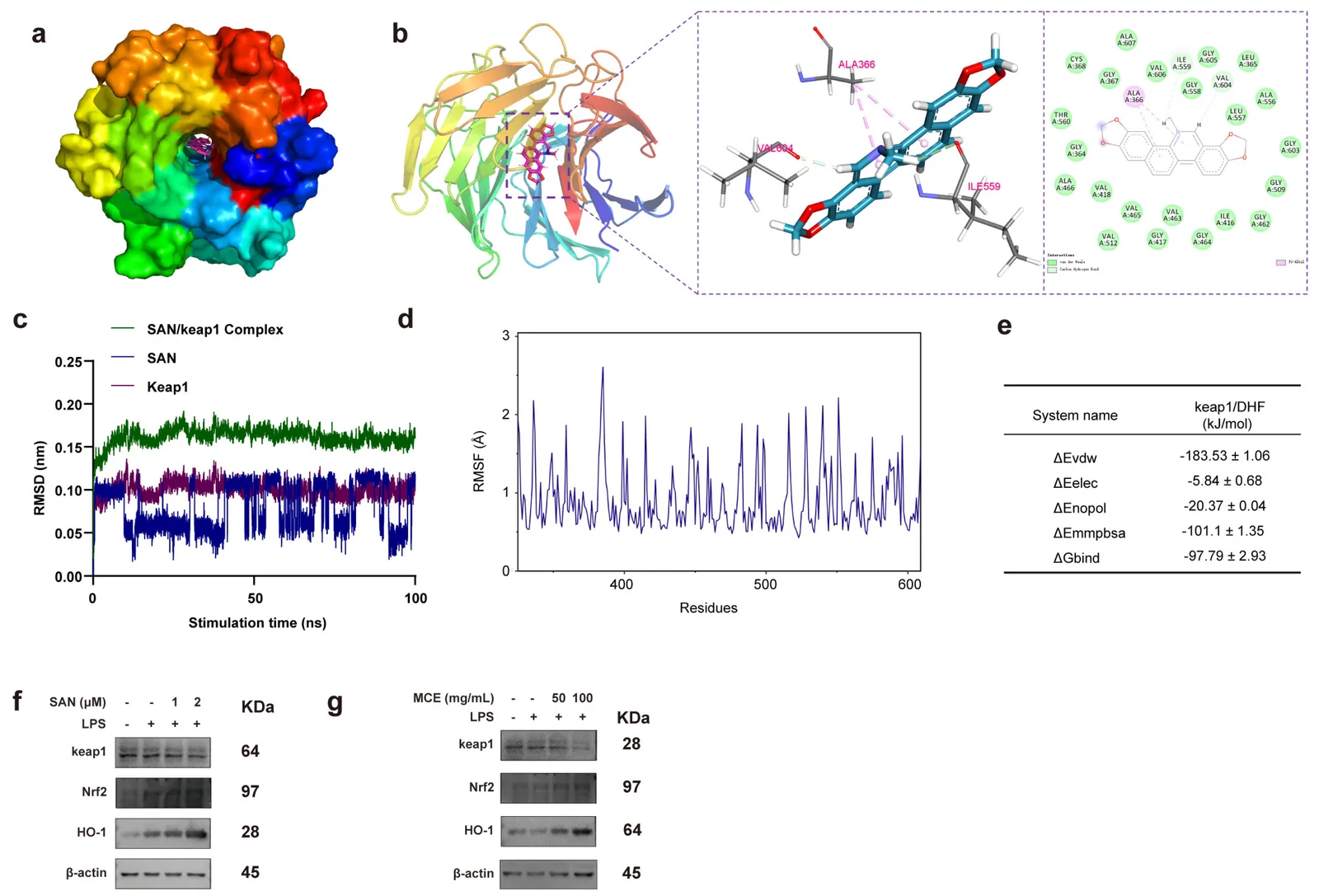
The effect of SAN on the Nrf2/HO-1 signaling pathway. (**a**,**b**) Molecular docking analysis of SAN with the keap1 protein (PDB: 4ZY3). (**c**) RMSD. (**d**) RMSF. (**e**) Binding free energies. (**f**) Effects of SAN on LPS-induced protein expression in the antioxidant signaling pathway in RAW 264.7 cells; whole-cell lysates were immunoblotted for keap1, Nrf2, and HO-1, with β-actin as the loading control. (**g**) Effects of MCE on LPS-induced protein expression of the same pathway in RAW 264.7 cells; Keap1, Nrf2, and HO-1 were detected by immunoblotting, and β-actin served as the loading control.

Considering the close association between inflammatory response and oxidative stress, we further investigated the cellular ROS accumulation upon LPS stimulation. It was worth noting that LPS drastically elevated the concentration of cellular ROS; nevertheless, SAN, but not CHE treatment, can significantly inhibit ROS accumulation (Figure 7o,p). Similarly, MCE showed a potent inhibitory effect against ROS production (Figure 7g,h). These results indicated the antioxidant potential of SAN. It has been widely reported that ligand binding to keap1 leads to the inhibition of Nrf2-mediated signaling, consequently preventing the subsequent oxidative stress induced by this pathway. Molecular docking was performed to explore the direct interaction between SAN and the keap1 protein. Our findings reveal that SAN establishes hydrogen bonding interactions with residues ILE559 and VAL604 within the binding pocket of Keap1 (Figure 8a,b). To further characterize the structural stability and conformational dynamics of the SAN-Keap1 interaction, molecular dynamics (MD) simulation analysis was conducted. Throughout the 100 ns simulation period, the RMSD values for the DHF-keap1 complex and the unbound keap1 protein remained stable within a narrow range of 1–2 Å (Figure 8c), suggesting that the binding of SAN did not induce any significant global conformational rearrangement. Meanwhile, the RMSF profiles exhibited relatively low values, with most residues along the protein sequence showing RMSFs below 2 Å (Figure 8d), which collectively indicates a rigid and well-maintained protein scaffold during the simulation. Furthermore, the binding free energy of the SAN-keap1 complex yielded a value of −97.79 ± 2.93 kJ/mol, which implies a favorable and stable binding affinity (Figure 8e). Furthermore, our results show that SAN and MCE effectively activated the expression of Nrf2/HO-1signaling pathway (Figure 8f,g). Therefore, it is reasonable to believe that SAN and MCE might inhibit the inflammatory response by activating the keap1/HO-1 signaling pathway.

## 4. Conclusions

In summary, this study demonstrated that MCE confers significant protective and therapeutic effects against 2.5% DSS-induced murine colitis. MCE supplementation ameliorated colitis by modulating gut microbiota composition (97.4% reduction in the relative abundance of pathogenic *Streptococcus* compared to the DSS group; *p* < 0.01) and suppressing M1 macrophage polarization (32.6% reduction in M1 macrophage compared to the DSS group). Furthermore, SAN, an active constituent of MCE, exerted similar anti-colitis effects. Mechanistically, both MCE and SAN inhibited the iNOS/NF-κB-mediated inflammatory response and activated the Nrf2/HO-1 antioxidant pathway. These findings indicate that MCE and its active monomer SAN may serve as potential preventive or alternative therapeutic agents for ulcerative colitis. However, the precise molecular mechanisms through which MCE and SAN ameliorate colitis via the gut microbiota–macrophage axis require further investigation.

## Data Availability

Data will be made available on request. The raw sequencing data have been deposited in the NCBI Sequence Read Archive (SRA) under BioProject number PRJNA1538986.

## Supplementary Materials

The following supporting information can be downloaded at: https://www.mdpi.com/article/10.3390/antiox15101344/s1. Figure S1. The reshapes the gut microbiota in CHE/SAN-induced mouse colitis. 16s rRNA sequencing of the colonic bacterial genome of C57BL/6J mice from the control, 2.5% DSS, DSS + SAN (1 mg/kg), DSS + CHE (1 mg/kg) (n = 4) were conducted. (a) Heatmap showing the top 10 microbial phylum based on UPGMA clustering. (b) The ternary phase diagram further showed the top 10 microbial genus based on UPGMA clustering. (b–e) The intestinal microbiota composition among experimental groups at the phylum (b) and the genus level (c) Akkemansia (d) and Dubosiella (e) among experimental groups at the genus level; Figure S2. The biosafety profile of SAN/CHE in mice. After the experimental period, H&E staining of the mice major organs from MCE-treated group was detected.

## Abbreviations

The following abbreviations are used in this manuscript:

DMSO	dimethyl sulfoxide
DSS	dextran sulfate sodium
H&E	hematoxylin and eosin
IBD	inflammatory bowel diseases
iNOS	inducible nitric oxide synthase
MCE	*Macleaya cordata* extract
DAI	disease activity index
CHE	chelerythrine
SAN	sanguinarine
