# Supplementary material for: Macleaya cordata Extract Ameliorates DSS-Induced Ulcerative Colitis via Inhibiting M1 Macrophages and Reshaping Gut Microbiota
Source: Antioxidants (Basel). 2026 Oct 16;15(10):1344. doi: 10.3390/antiox15101344 (PMC13701535; doi:10.3390/antiox15101344)
Supplement: Supplementary file 1 [file antioxidants-15-01344-s001.zip › antioxidants-4603957-supplementary.pdf]

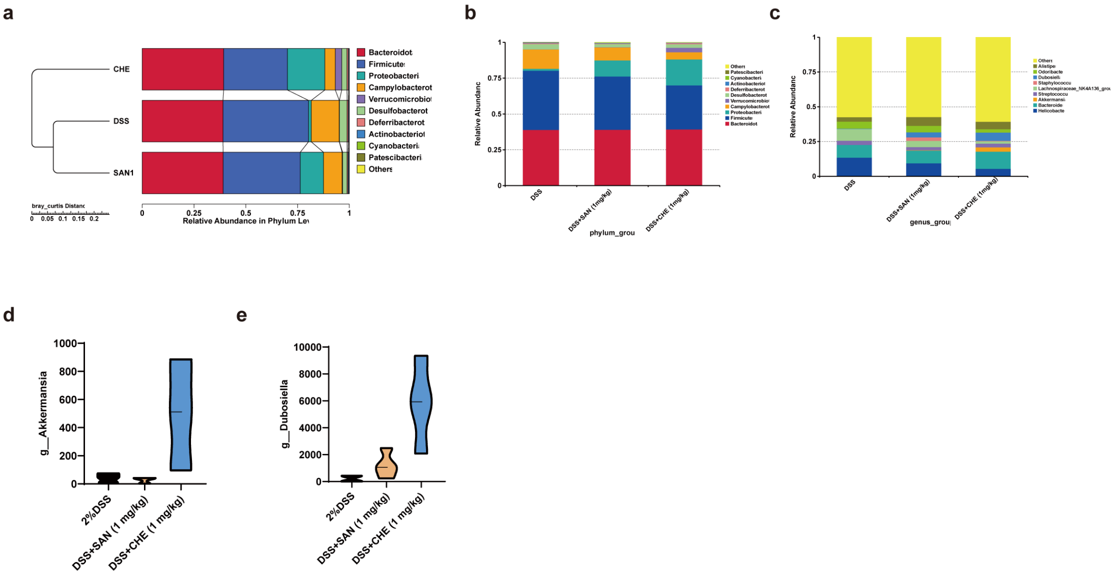

Figure S1 The reshapes the gut microbiota in CHE/SAN-induced mouse colitis. 16s rRNA sequencing of the colonic bacterial genome of C57BL/6J mice from the control, 2.5% DSS, DSS +SAN (1 mg/kg), DSS +CHE (1 mg/kg)(n = 4) were conducted. (**a**) Heatmap showing the top 10 microbial phylum based on UPGMA clustering. (**b**) The ternary phase diagram further showed the top 10 microbial genus based on UPGMA clustering. (**b-e**) The intestinal microbiota composition among experimental groups at the phylum (**b**) and the genus level (**c**) Akkemansia (**d**) and Dubosiella (**e**) among experimental groups at the genus level.

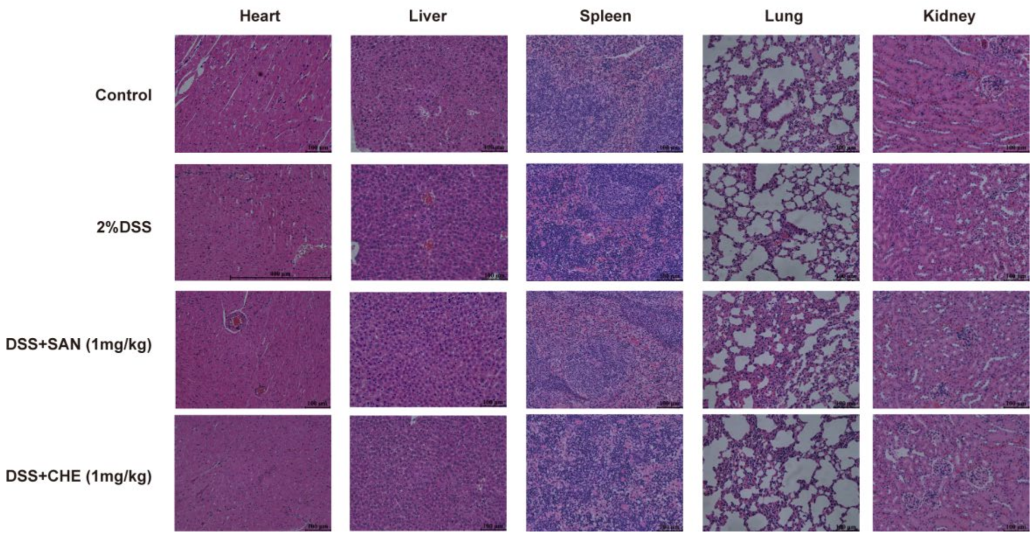

Figure S2 The biosafety profile of SAN/CHE in mice. After the experimental period, H&E staining of the mice major organs from MCE-treated group was detected.
